# Genetic diversity of *Trypanosoma cruzi* in individuals with chronic Chagas disease in the Northern Minas Gerais and Vale do Jequitinhonha regions, Minas Gerais, Brazil

**DOI:** 10.1371/journal.pntd.0013627

**Published:** 2025-10-15

**Authors:** Emanuelle de Moura Santos Xavier, Paula Finamore Araujo, Jenifer Raiane Silva Pedras, Thainara da Silva Gonçalves, Núbia Nunes de Souza, Ariela Mota Ferreira, Léa Campos de Oliveira-da Silva, Ester Cerdeira Sabino, Maria do Carmo Pereira Nunes, Antonio Luiz Pinho Ribeiro, Otacilio C. Moreira, Thallyta Maria Vieira

**Affiliations:** 1 Health Science Programme, State University of Montes Claros, Montes Claros, Brazil; 2 Molecular Virology and Parasitology Laboratory, Oswaldo Cruz Institute, Oswaldo Cruz Foundation, Rio de Janeiro, Brazil; 3 Laboratorio de Genetica de Microorganismos Aplicado a Medicina (LIM46), Hospital das Clínicas HCFMUSP, Faculdade de Medicina, Universidade de São Paulo, São Paulo, SP, Brazil; 4 Federal University of Minas Gerais, School of Medicine, Department of Internal Medicine, Minas Gerais, Brazil; San Diego State University, UNITED STATES OF AMERICA

## Abstract

Chagas disease, caused by the hemoflagellate parasite *Trypanosoma cruzi*, is a major public health problem in Latin America due to its high prevalence and significant morbidity and mortality. The *T. cruzi* is classified into seven distinct genetic groups (TcI-TcVI) and TcBat, know as *Discrete Typing Units* (DTUs). Understanding DTU diversity is essential for improving diagnostic and therapeutic strategies and strengthening epidemiological surveillance. This study aimed to investigate the genetic diversity of *T. cruzi* in individuals with chronic Chagas disease from endemic municipalities in Northern Minas Gerais and the Jequitinhonha Valley, Brazil. It also evaluated associations between DTUs and age, parasitic load, antibody levels, and cardiac disease severity in participants from the SaMi-Trop cohort. Molecular typing was performed using conventional multilocus PCR directly from peripheral blood samples of individuals with the chronic cardiac form. Of the 80 patients included in the study, *T. cruzi* genotyping was performed in 37 samples (46.25%). Among the samples in which a single DTU was identified, TcI and TcVI presented equal frequencies (n = 5, 13.51% each). TcII was identified in four samples (n = 4, 10.81%), while TcV was identified in three samples (n = 3, 8.10%). Furthermore, three individuals presented mixed infections: TcV + TcVI, TcI + TcV + TcII/TcVI, and TcV + TcII. The highest frequency was observed for TcII/TcVI (n = 17, 45.94%), a classification that does not represent mixed infection. For statistical analysis, TcII/TcVI samples were classified as TcII. No significant differences were found between the DTUs and age or parasitic load. However, a wide variation in the average parasitic load was observed among individuals, ranging from 0.06 to 667 parasite equivalents/mL. An increase in anti-*T. cruzi* antibody titers was also observed. The findings of this study demonstrate the genetic diversity of the parasite in individuals with chronic cardiac Chagas disease and its distribution in a highly endemic region.

## Author summary

Even 115 years after its discovery, Chagas disease remains an endemic and neglected disease. Understanding the genetic diversity of *Trypanosoma cruzi* is essential to improve diagnosis and control strategies. This study investigated the genetic diversity of *T. cruzi* in individuals with the chronic cardiac form of the disease, from endemic regions of the North and Jequitinhonha Valley, in Minas Gerais. Molecular typing revealed that, for samples with a single identified DTU, TcI and TcVI exhibited equal frequencies, followed by TcII and TcV. Mixed infections were also identified. The highest frequency was observed for TcII/TcVI, a classification that does not represent mixed infection. No significant differences were found between DTUs and age or parasitic load, while an increase in anti-*T. cruzi* antibody titers was detected. These findings reinforce the importance of molecular characterization of *T. cruzi* to understand the ecoepidemiology of Chagas disease, especially in regions with high diversity of DTUs and occurrence of mixed infections. This information is essential for the development of more effective diagnostic, treatment and control strategies.

## Introduction

Within tropical ecosystems and rural and urban communities, a persistent challenge echoes: Chagas disease (CD). Caused by the hemoflagellate parasite *Trypanosoma cruzi* [1], the infection transcends geographic and temporal boundaries, remaining a scientific and public health dilemma. Early detection of CD is often hindered by the absence of clinical symptoms, as approximately 70% of individuals with chronic CD are asymptomatic (indeterminate form), while 30% exhibit cardiac and digestive manifestations, which can be potentially fatal [2,3]. Furthermore, it is estimated that between 6 and 7 million people worldwide are currently infected with the parasite [4].

*T. cruzi* exhibits remarkable genetic heterogeneity, classified into seven distinct lineages: TcI to TcVI, as well as TcBat. These lineages are commonly referred to as “*Discrete Typing Units*” (DTUs) [5]. The correlation between *T. cruzi* DTU patterns and the severity of CD has resulted in a general, though not definitive, association between TcI and oral transmission or chronic cardiac forms, while TcII, TcV, and TcVI have been linked to more severe chronic cardiac or digestive manifestations. On the other hand, TcIII, TcIV, and especially TcBat are rarely associated with human infections [3,6,7]. However, Monteiro et al. [8] reported that both TcI and TcIV circulated simultaneously in areas of the Western Brazilian Amazon and were capable of infecting humans, resulting in cases of acute infection. It is noteworthy that TcIV was identified as the main strain related to human infections in this region, occurring in outbreaks both as single infections and in co-infections involving different haplotypes.

The macro-regions of health in Northern Minas and the Jequitinhonha Valley have the highest vulnerability indices for chronic CD in Brazil, as well as high risk for vector transmission of Chagas disease [9,10]. Recent reports of infected triatomines in the urban area of a municipality in Northern Minas Gerais raise concerns about the risk of *T. cruzi* transmission to humans and domestic animals [11]. These data, reflecting the population’s susceptibility to the disease, are exacerbated by limited access to healthcare services and low case detection, impacting the population’s quality of life [9]. Additionally, there are shortcomings in CD surveillance and vector control efforts, highlighting the urgent need for more specific interventions in these regions [12].

Therefore, it is crucial to deepen our understanding of the ecoepidemiology of Chagas disease to enhance diagnostic and therapeutic approaches, as well as strengthen prevention and control strategies. In this context, the present study aims to investigate the genetic diversity of *Trypanosoma cruzi* in individuals with the chronic form of Chagas disease from 20 endemic municipalities in the Northern Minas and Jequitinhonha Valley regions, in Minas Gerais, Brazil, and verify if there is a correlation between the *T.cruzi* genotypes with the individuals’ age, parasitic load, antibody titers, and the severity of cardiac disease.

## Methods

### Ethics, consent, and permissions

In compliance with the ethical principles of Resolution 466/2012 of the Brazilian National Health Council, which regulates research involving human subjects, this study was approved by the National Research Ethics Commission (CONEP, Approval No. 179.685/2012) and the Research Ethics Committee of the University of São Paulo School of Medicine (Approval Nº. 042/2012). All participants provided informed consent by signing the Informed Consent Term or Informed Consent Form (*Termo de Consentimento Livre e Esclarecido* - TCLE).

### Study design

This is a longitudinal study utilizing epidemiological, clinical, and laboratory data from the SaMi-Trop study (Research Center for Biomarkers in Neglected Tropical Diseases of São Paulo/Minas Gerais). SaMi-Trop is a prospective cohort study involving individuals with Chagas disease, conducted across 21 municipalities in the northern region of Minas Gerais and the Jequitinhonha Valley, Brazil. The study’s baseline data were collected during 2013–2014, with the first follow-up visit occurring in 2015–2016 and the second follow-up conducted between 2019 and 2021 [13,14].

### Study population

**Fig 1:**
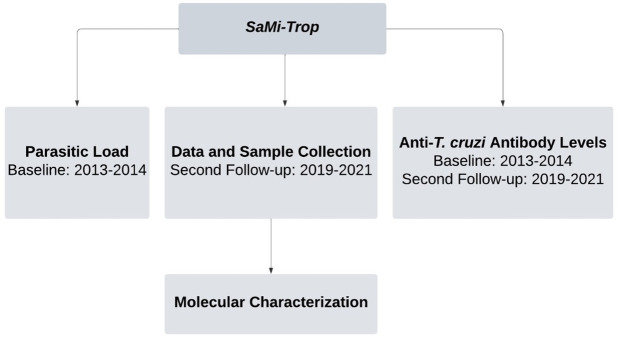
Flowchart of the study data collection process.

For mapping and recruiting patients with CD in the studied regions, the database of the Minas Gerais Telecare Network (RTMG), linked to Primary Health Care, was used. Individuals aged ≥18 years, who self-reported CD and presented alterations in the electrocardiogram (ECG) performed between 2011 and 2012 by RTMG were included. Additional details on eligibility criteria, sample size and operational procedures are available in previous publications [13–15]. Participants were selected by convenience sampling during recruitment; therefore, no sample size calculation was performed. Approximately 200 patients with positive serology for *T. cruzi* were recruited. Of these, 80 presented positive results in qPCR. Peripheral blood samples were collected during follow-up visits and sent for genotyping to identify *T. cruzi* DTUs. Parasite burden was assessed at baseline through real-time quantitative PCR (qPCR), targeting the satellite DNA of *T. cruzi*, according to Piron et al. [16]. Levels of anti-*T. cruzi* antibodies were evaluated both at baseline and during the second follow-up using commercial kits with different antigenic preparations (Architect Chagas, Abbott Laboratories, Wiesbaden, Germany), following the manufacturer’s instructions [17]. Epidemiological and clinical data, along with the collection of peripheral blood samples for genotyping, were obtained during the second follow-up phase of the study (Fig 1).

### Samples from individuals

All participants reported not receiving etiological treatment at the time of the study. Venous blood samples (10 mL) were collected from each individual and immediately mixed, in a 1:1 ratio, with 6M guanidine hydrochloride/0.2 M EDTA solution (pH 8.0). They were then stored at 4°C until further processing.

### DNA extraction

DNA was extracted from 300 μL of venous blood in guanidine-EDTA using the “High Pure PCR Template Preparation Kit” (Roche, Mannheim, Germany), following the protocol recommended by Ramírez et al. [18]. In the final step of the protocol, DNA was eluted in 100 μL of elution buffer and stored at -20°C until use.

### Reference samples of Trypanosoma cruzi

As a panel of positive controls (DNA extracted from reference strains of *T. cruzi*), epimastigote forms of *T. cruzi* cultivation classified as DTUs TcI to TcVI were used. These included the following clones/strains: Dm28c (TcI), Y (TcII), 3663 (TcIII), 4167 (TcIV), LL014 (TcV), and CL Brener (TcVI), obtained from the Protozoan Collection of the Oswaldo Cruz Foundation (COLPROT/Fiocruz).

### Trypanosoma cruzi genotyping

**Fig 2:**
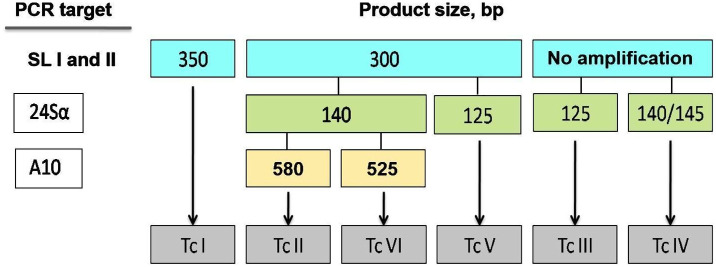
Flowchart for *T. cruzi* genotyping directly from clinical samples. The figure displays the expected amplicon sizes for each PCR target in base pairs (bp). SL-IR I and II: spliced leader gene regions, 24Sα rDNA: divergent D7 domain of the 24Sα gene, A10: nuclear A10 fragment. Source: [19].

The genotyping of *T. cruzi* into DTUs I to VI was performed using multilocus conventional PCR [19]. Genotypes were identified based on the combined analysis of PCR product profiles for each target, using the following molecular markers (Fig 2): Spliced Leader intergenic region I and II: Primers TCC, TC1, and TC2 were used to differentiate TcI (350 bp), TcII, V, and VI (300 bp), and TcIII and IV (non-amplified). Variable D7 domain of the 24Sα ribosomal RNA subunit gene: Semi-nested PCR was performed using primers D75 and D76 (first round) and D76 and D71 (second round) to distinguish TcII and VI (140 bp), TcIII (125 bp), TcIV (140/145 bp), and TcV (125 or 125 + 140 bp). A10 nuclear fragment region: Semi-nested PCR using primers Pr1 and P6 (first round) and Pr1 and Pr3 (second round) differentiated TcII (690/580 bp) and TcVI (630/525 bp).

By convention, the representation of *T. cruzi* followed by the symbol (/) indicates that the sample may be infected with any of the mentioned DTUs. In these cases, (/) signifies that the genotyping criteria used were not able to identify a specific DTU among those presented.

The amplification reactions were performed using the Veriti Thermal Cycler (Applied Biosystems) as follows: 5 μL of extracted DNA were added to 12.5 μL of 2X GoTaq Green Master Mix (Promega, Madison, USA), containing GoTaq DNA polymerase, buffer (pH 8.5), 400 μM of each dNTP, and 3 mM of MgCl2, along with 1.25 μL of each primer (stock solutions: 25 μM for the SL-IR target, 10 μM for the 24Sα and A10 targets) and 5 μL of ultrapure water. The PCR products (25 μL) were separated by agarose gel electrophoresis (2% and 3% w/v, 90V), stained with 0.1X GelRed (Biotium), and visualized under ultraviolet light. A 100 bp molecular weight marker (Ludwig) was used to estimate the size of the amplified products.

### Variables

The genotyping results were used as the outcome (dependent variable), categorized as follows: TcI, TcII, TcV, TcVI, TcII/TcVI, TcV + TcII/TcVI, TcI + TcV + TcVI, and TcV + TcII. Independent variables included municipality, age, gender, parasite burden, antibody levels, and severity profile. Patient age was calculated based on their date of birth and categorized into two groups: ≤ 60 years and >61 years. Gender was categorized as male or female. Regarding severity profile, parameters were classified as follows: Electrocardiogram (ECG) changes: categorized as normal/minor alterations or major alterations. Functional class: classified into I, II, III, and IV. B-type natriuretic peptide (BNP): categorized by age as normal or altered [20]. Ejection fraction: categorized as >50% (normal) or ≤50% (altered) [21]. These data were obtained from electrocardiograms, laboratory tests, echocardiograms, and the New York Heart Association (NYHA) Functional Classification.

### Statistical analysis

A descriptive analysis was conducted, presenting simple frequencies (n) and relative frequencies (%) for categorical variables. Subsequently, bivariate analysis was performed using Fisher’s exact test, considering only associations with a significance level of up to 5% (p < 0.05). The normal distribution of continuous data was verified using the Shapiro-Wilk test. The non-parametric Kruskal-Wallis test was employed for comparative analysis, with significance set at p < 0.05. To assess the variation in anti-*T. cruzi* antibody titers between baseline and follow-up, the paired-sample t-test was applied, considering a significance level of 5%. All analyses were conducted using Predictive Analytics Software - IBM SPSS Statistics version 18.0.

### Spatial distribution

Georeferencing of DTU cases was conducted using the geographic meshes of municipalities in northern Minas Gerais and the Jequitinhonha Valley, extracted from the publicly accessible cartographic database of the Brazilian Institute of Geography and Statistics (Instituto Brasileiro de Geografia e Estatística - IBGE) (https://www.ibge.gov.br/geociencias/downloads-geociencias.html). Map construction was performed using QGIS software, version 3.34.7 (https://www.qgis.org/).

## Results

The study included 80 individuals with the cardiac form of chronic Chagas disease, aged between 34 and 96 years, with a predominance of females (n = 59, 73.8%). Molecular typing of DTUs was successfully identified in 46.25% (37) of blood samples from individuals aged between 34 and 91 years, of whom 27 were female (73%) (S1 Table).

Regarding genotypic characterization, a higher frequency of TcII/TcVI was observed, identified in 45.94% of cases (17/37). The DTUs TcI and TcVI presented frequencies equal to 13.51% (5/37 each), while TcII was detected in 10.81% (4/37) of individuals. In addition, 8.10% of individuals were infected with TcV (3/37) and three cases presented mixed infections (more than one DTU), namely, TcV + TcVI, TcI + TcV + TcII/TcVI and TcV + TcII. It is noteworthy that TcV was present in all cases of mixed infection (S1 Table). For statistical analysis, samples whose profile could not be differentiated (TcII/TcVI) were considered as TcII.

**Fig 3:**
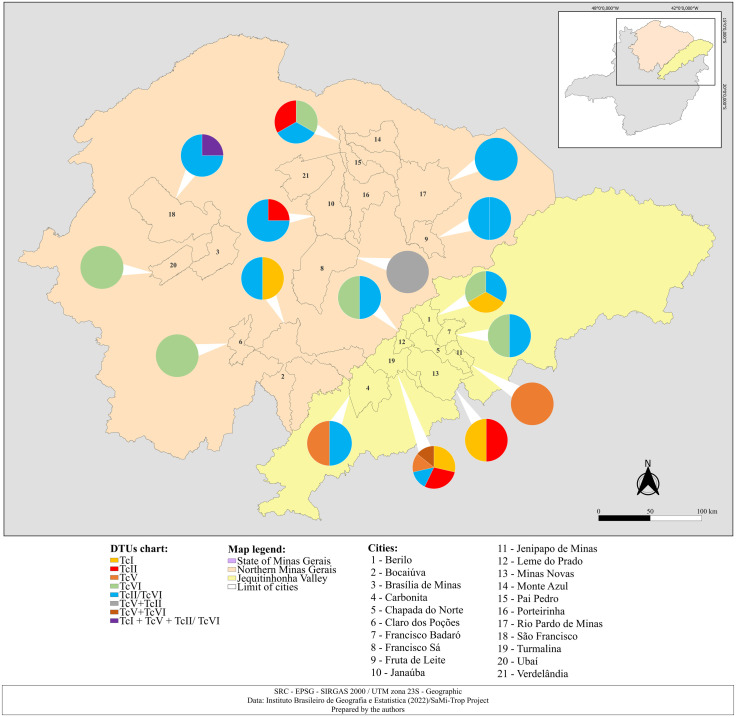
Geographic distribution of *Trypanosoma cruzi* DTUs in samples from individuals from the Northern and Jequitinhonha Valley regions (n = 37), Minas Gerais, Brazil, 2024.

In terms of the distribution of *T. cruzi* DTUs identified in the northern region of Minas Gerais and the Jequitinhonha Valley, TcI as a single infectious DTU was identified in five different municipalities, TcII in four, TcV in three, TcVI in five, and an unresolved TcII/TcVI profile was identified in 11 of the 20 distinct municipalities analyzed. Furthermore, mixed infections were observed in three municipalities: Turmalina, São Francisco, and Francisco Sá (Fig 3). The municipality of Turmalina stood out for concentrating 18.9% of the samples positive for *T. cruzi*, representing the highest proportion among the municipalities analyzed. In addition, Turmalina also presented a greater diversity of DTUs among these municipalities, with the identification of TcI, TcII, TcV, TcII/TcVI and mixed infection (TcV + TcVI), providing a complex transmission dynamic of the parasite in the region (Fig 3).

The majority of DTUs (67.6%) were identified in individuals over 61 years of age (Table 1), although no statistically significant difference was observed between age and DTUs. Additionally, patients over 61 years presented the highest mean parasite burden (57.73 Eq. parasites/mL).

**Table 1:** Comparison between municipalities, gender, and age group of the study individuals according to the identified DTUs (n = 37).

		DTUs								p-value
	n (%)	TcI	TcII	TcV	TcVI	TcII/TcVI	TcV+ TcVI	TcI + TcV +TcII/TcVI	TcV+TcII	
**Municipalities**										
Berilo	3 (8.1)	1 (20)	0 (0)	0 (0)	1 (20)	1 (5.9)	0 (0)	0 (0)	0 (0)	0.921
Bocaiuva	2 (5.4)	1 (20)	0 (0)	0 (0)	0 (0)	1 (5.9)	0 (0)	0 (0)	0 (0)	
Carbonita	2 (5.4)	0 (0)	1 (10)	1 (33.3)	0 (0)	1 (5.9)	0 (0)	0 (0)	0 (0)	
Claro dos Poções	1 (2.7)	0 (0)	0 (0)	0 (0)	1 (20)	0 (0)	0 (0)	0 (0)	0 (0)	
Francisco Badaró	2 (5.4)	0 (0)	0 (0)	0 (0)	1 (20)	1 (5.9)	0 (0)	0 (0)	0 (0)	
Francisco Sá	1 (2.7)	0 (0)	0 (0)	0 (0)	0 (0)	0 (0)	0 (0)	0 (0)	1 (100)	
Fruta de Leite	2 (5.4)	0 (0)	0 (0)	0 (0)	0 (0)	2 (11.8)	0 (0)	0 (0)	0 (0)	
Janaúba	3 (8.1)	0 (0)	1 (25)	0 (0)	0 (0)	2 (11.8)	0 (0)	0 (0)	0 (0)	
Jenipapo de Minas	1 (2.7)	0 (0)	0 (0)	1 (33.3)	0 (0)	0 (0)0	0 (0)	0 (0)	0 (0)	
Leme do Prado	2 (5.4)	0 (0)	0 (0)	0 (0)	1 (20)	1 (5.9)	0 (0)	0 (0)	0 (0)	
Minas Novas	2 (5.4)	1 (20)	1 (25)	0 (0)	0 (0)	0 (0)	0 (0)	0 (0)	0 (0)	
Pai Pedro	3 (8.1)	0 (0)	1 (25)	0 (0)	1 (20)	1 (5.9)	0 (0)	0 (0)	0 (0)	
Rio Pardo de Minas	1 (2.7)	0 (0)	0 (0)	0 (0)	0 (0)	1 (5.9)	0 (0)	0 (0)	0 (0)	
São Francisco	4 (10.8)	0 (0)	0 (0)	0 (0)	0 (0)	3 (17.6)	0 (0)	1 (100)	0 (0)	
Turmalina	7 (18.9)	2 (40)	1(25)	1 (33.3)	0 (0)	2 (11.8)	1 (100)	0 (0)	0 (0)	
Ubaí	1 (2.7)	0 (0)	0 (0)	0 (0)	0 (0)	1 (5.9)	0 (0)	0 (0)	0 (0)	
**Gender**										
Male	10 (27)	3 (60)	2 (50)	0 (0)	1 (20)	4 (23.5)	0 (0)	0 (0)	0 (0)	0.598
Female	27 (73)	2 (40)	2 (50)	3 (100)	4 (80)	13 (76.5)	1 (100)	1 (100)	1 (100)	
**Age**										
≤ 60 years	12 (32.4)	1 (20)	1 (25)	1 (33.3)	1 (20)	6 (35.3)	0 (0)	1 (100)	1 (100)	0.767
≥ 61 years	25 (67.6)	4 (80)	3 (75)	2 (66.7)	4 (80)	11 (64.7)	1 (100)	0 (0)	0 (0)	

**Fig 4:**
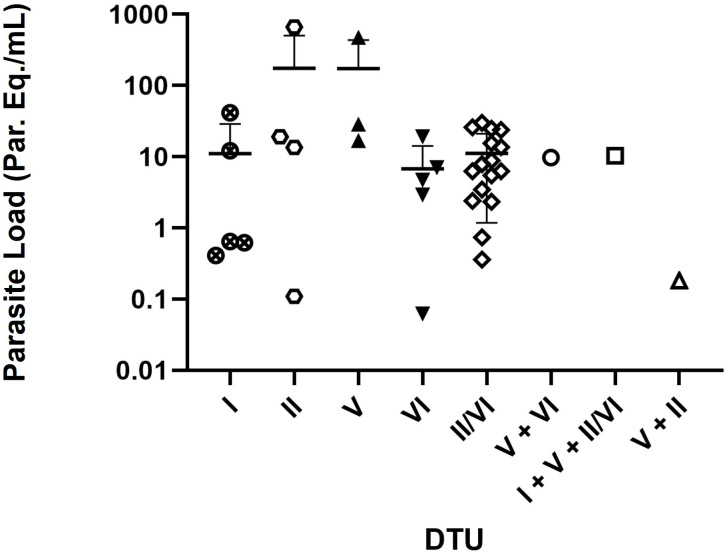
Comparison of parasite burden in individuals with chronic Chagas disease by identified DTUs (n = 37).

When comparing the parasite load between the different DTUs, it was observed that individuals infected with TcV and TcII/VI presented parasitemia means of 174.00 Eq. parasites/mL and 57.28 Eq. parasites/mL, respectively. Despite these higher means, the difference in parasitemia levels between TcV and TcII/VI DTUs was not statistically significant (Fig 4).

**Fig 5:**
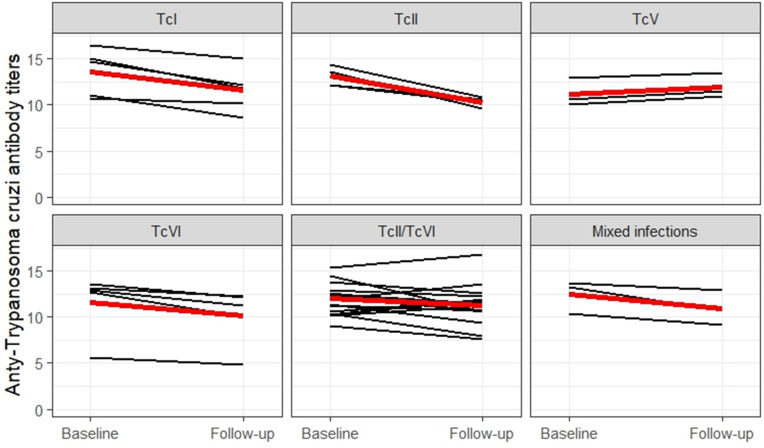
Comparison of anti-*Trypanosoma cruzi* antibody titers in individuals with chronic Chagas disease at baseline (2013/2014) and follow-up (2019/2021), distributed by the identified DTUs (n = 37). The black line represents the individual trajectory of each participant between periods, while the red line represents the genotype mean [17].

When comparing the titers of anti-*Trypanosoma cruzi* antibodies produced by individuals between baseline and follow-up, a mean increase was observed only in the titers of those identified with TcV (Fig 5), while in the other individuals, there was a reduction in antibody levels over time.

The majority of the analyzed samples did not show a cardiac severity profile. However, 60% (3/5) of individuals infected with TcI exhibited major ECG alterations and were classified in functional class IV. Individuals infected with TcV also displayed major ECG alterations, with a frequency of 66.7% (2/3), and were classified in functional class III. Among the three individuals with mixed infection, all showed altered BNP levels, with two exhibiting major ECG alterations and two being classified in functional class III (Table 2).

**Table 2:** Cardiac severity profile of individuals with chronic Chagas disease from the Northern and Jequitinhonha Valley regions, Minas Gerais, Brazil, according to the DTUs (n = 37).

Severity profile parameters	Frequency	TcI	TcII	TcV	TcVI	TcV + TcVI	TcV + TcII	TcI + TcV + TcII/TcVI	p-value
	n (%)								
ECG changes*									
Normal/Minor changes	20 (55.6)	2 (40)	13 (65)	1(33.3)	3 (60)	1 (100)	0	0	0.555
Major changes	16 (44.4)	3 (60)	7 (35)	2 (66.7)	2 (40)	0	1 (100)	1 (100)	
**Functional class**									
Class I	9 (24.3)	1 (20)	6 (28.6)	1(33.3)	1(20)	0	0	0	0.406
Class II	7 (18.9)	0 (0)	6 (28.6)	0 (0)	0 (0)	0	1 (100)	0	
Class III	10 (27)	1 (20)	3 (14.3)	2 (66.7)	2(40)	1 (100)	0	1 (100)	
Class IV	11 (29.7)	3 (60)	6 (28.6)	0 (0)	2 (40)	0	0	0	
**BNP**									
Unchanged	29 (78.4)	5 (100)	20 (95.2)	3 (100)	1 (20)	0	0	0	**<0.001**
Changed	8 (21.6)	0	1 (4.8)	0	4 (80)	1 (100)	1 (100)	1 (100)	
Ejection fraction*									
Normal	33 (91.7)	5 (100)	17 (85)	3 (100)	5 (100)	1 (100)	1 (100)	1 (100)	1.000
Changed	3 (8.3)	0	3 (15)	0	0	0	0	0	

* Variation of n = 37 due to missing information.

**Footnote:**

TcII/TcVI is presented separately for descriptive purposes only. The TcII column (now including all TcII/TcVI) represents the extreme scenario in the sensitivity analysis for uncertainty assessment in classification, demonstrating that the statistical conclusion remained robust despite the different allocation.

## Discussion

This study revealed a broad diversity of *T. cruzi* DTUs among individuals with chronic Chagas disease in the endemic municipalities of Northern Minas Gerais and the Jequitinhonha Valley, greater than previously reported. Although *T. cruzi* infection does not exhibit a gender preference [22], the predominance of females and the mean age of 66.5 years among the study participants reflect the expected epidemiological profile for chronic Chagas disease [23,24].

A study conducted in Northern Minas Gerais identified an overall prevalence of the disease exceeding 9%, with individuals aged 4–14 years showing a prevalence of 0.8%, whose mothers had negative serology. These results suggest the continuation of active transmission of the disease through vectors [25]. Another study conducted in a municipality in Northern Minas registered the occurrence of *T. cruzi* infected triatomine species in urban areas. The presence of these species represents a significant public health issue, as it suggests a risk of parasite transmission to humans and domestic animals [11]. However, the Jequitinhonha Valley remains one of the areas with the highest vulnerability to chronic Chagas disease [9].

Our study, as well as others [24–26], has identified an increased frequency of elderly individuals with CD, which appears to reflect a series of interconnected factors. Among them, the success of vector control campaigns — such as the elimination of *Triatoma infestans* — and transfusion transmission stand out, which resulted in a significant reduction in the incidence of acute cases in Brazil [24,26]. However, a high rate of underdiagnosis still persists, and many infected individuals are unaware of their condition [25]. Despite the occurrence of native triatomines in households, the CD control program is disjointed, with discontinuous actions [12]. Furthermore, there is no systematic investigation of CD in routine prenatal care [27], and many health professionals are still unaware of the disease or do not consider it in clinical suspicion, which compromises early diagnosis and treatment [28,29].

In this study, a higher frequency of TcII/TcVI (45.94%) was observed among the samples analyzed. This finding is in agreement with the results of Tavares de Oliveira *et al*. [30], who, among 330 positive patients, managed to genotype 175 samples, of which 23 (13.1%) presented the TcII/TcVI profile in Southeast Brazil. The distinction between TcII and TcVI DTUs was not possible in some cases because the A10 molecular target did not amplify, even after repeated PCR attempts. Although this target is known for its high sensitivity, amplification failures can occur due to the low parasitic load of the individuals. These results are consistent with the findings of Rodrigues-dos-Santos et al. [31] and Oliveira et al. [30], who also reported infections by TcII/TcVI without being able to differentiate the DTUs. Furthermore, these findings reinforce previous investigations into the frequency of TcII/TcVI in various regions of Brazil, especially associated with the domestic transmission cycle of Chagas disease [31,32].

Among the samples in which a single DTU was identified, TcI and TcVI had equal frequencies (13.51%). Souza et al. [33] identified the presence of TcI in 9.52% (2/21) of individuals with chronic Chagas disease from the interior of São Paulo State. In addition, Tavares de Oliveira et al. [30] identified the presence of the TcI genotype in three individuals in the Southeast region of Brazil. Complementarily, Cunha et al. [34], in a systematic review, observed that, in Northern Brazil (states of Amapá, Tocantins, Pará and Amazonas), 25.2% of human infections by *T. cruzi* were attributed to the TcI genotype, transferring a geographic distribution of this genotype throughout the country. In the Southeast region of Brazil, TcI was detected in 3.3% of individuals, with most cases originating from the state of Minas Gerais. Meanwhile, TcVI is strongly associated with the domestic cycle of Chagas disease in some regions of the Southern Cone [3]. This DTU has been reported in human infections in the Chaco region, northern Argentina, Chile [35], and southern Brazil, where 11 strains isolated from individuals involved in an oral transmission outbreak of *T. cruzi* in Santa Catarina were characterized [36]. Additionally, it was identified in an endemic area in Minas Gerais, in the Jequitinhonha Valley, where 63 samples were genotyped, and 11.1% presented TcVI [37].

Regarding the TcII genotype, 10.81% of infected individuals were identified, which is in line with previous studies that highlight the predominance of this DTU in endemic regions of Brazil [30,31,38]. Lineage II is widely associated with human infections, especially in the South and Southeast regions of the country, and is present in both domestic and sylvatic transmission cycles of CD. Furthermore, TcII has been frequently associated with severe clinical manifestations, including cardiac and digestive forms of the disease [3,7,30,39].

Regarding TcV, 8.1% of the individuals were infected. Until now, this DTU had not been reported in human infections in the studied regions; however, this genotype has been detected in the Brazilian Amazon [40]. Subsequent studies using various genotyping techniques corroborated the predominance of TcV in individuals with different cardiac and/or digestive pathologies [3,41,42]. The presence of TcV is commonly linked to vectors and humans in the domestic infection cycle, rarely observed in the sylvatic cycle in various Latin American regions [3].

Three samples presented mixed infections: one with TcV + TcVI, another with TcI + TcV + TcII/TcVI, and the last with TcV + TcII. This finding is consistent with the study of Abras et al. [43], who identified mixed infections in two individuals, one with TcV + TcII/TcVI and the other with TcV + TcII, among 20 individuals analyzed in the Barcelona region, Spain. The DTUs TcV, TcII, and TcVI seem to be concentrated in Central and Southern South America, primarily restricted to domestic transmission cycles. Furthermore, the study of Oliveira et al. [44] also detected the presence of these infections in 33.7% (n = 26) of 77 individuals from Bolivia. This is the first time that a mixed infection by TcI + TcV + TcII/TcVI is identified in a single sample from an individual in Northern Minas.

The species *Triatoma melanica* was identified in a study conducted in Northern Minas Gerais. Of the 63 *T. cruzi*-positive samples, 56 were genotyped. The TcI genotype was the most prevalent, representing 55% (31/56), followed by TcIII with 20% (11/56) and TcII with 7% (4/56). Additionally, mixed infections by TcI + TcIII were detected in 18% (10/56) of the samples [45]. It is essential to adopt an integrated view of the environment, recognizing that humans are not isolated, and that *T. cruzi* reservoirs can connect domestic and sylvatic transmission cycles [46]. In this context, the spread of different *T. cruzi* DTUs suggests an interconnection between transmission cycles.

The distribution of *T. cruzi* DTUs in the northern regions of Minas Gerais and the Jequitinhonha Valley reveals a specific complexity in the epidemiology of CD. The presence of vectors infected with different DTUs in geographically close municipalities may indicate the existence of multiple transmission cycles, including sylvatic transmission, or may suggest that some of the infected individuals may have been exposed to the parasite in forested or adjacent rural areas, where sylvatic transmission is present. This distribution pattern highlights not only the genetic diversity of the parasite, but also suggests relevant implications for disease control and prevention strategies. The identification of circulating DTUs in certain regions may support more accurate therapeutic decisions and contribute to the optimization of treatment protocols. Furthermore, understanding the transmission routes associated with these DTUs deepens knowledge about the ecoepidemiology of Chagas disease, guiding the implementation of more effective surveillance actions.

Individuals infected with TcV and TcII/VI had high mean parasitemia levels (174.00 Eq. parasites/mL and 57.28 Eq. parasites/mL, respectively). However, this difference was not statistically significant. This finding is consistent with Rodrigues-dos-Santos et al. [31], who also reported higher parasite loads in individuals infected with TcII/TcVI, although no statistically significant association was found between DTU and parasitemia levels. This pattern may be related to the presence of comorbidities or advanced age, factors that may favor immunosuppression and reactivation of Chagas disease. Our data also reinforce this observation, as older individuals had the highest mean parasitic loads.

When analyzing anti-*Trypanosoma cruzi* antibody titers, a significant increase was observed only in individuals infected with TcV. According to Nunes et al. [21], elevated levels of these antibodies are associated with higher mortality in individuals with Chagas disease. Additionally, the study indicated that individuals with higher antibody titers had a threefold higher incidence of cardiomyopathy. These findings highlight the role of anti-*T. cruzi* antibodies as markers of poor prognosis and greater risk of cardiac complications, emphasizing the importance of monitoring these titers in the risk assessment and clinical management of these individuals [47]. However, it is important to note that DTU identification in our study was performed only at the follow-up. Therefore, we cannot exclude the possibility that a different DTU could have been present at the time of recruitment (study baseline), but was not detected later during follow-up.

The analysis of the cardiac severity profile reveals significant information in individuals infected with different DTUs, specifically TcI and TcV, as well as mixed infections. Most samples did not show a severe cardiac profile, but the data indicate that a significant proportion of individuals showed ECG alterations and functional classifications that deserve attention.

Among individuals infected with TcI, 60% exhibited significant ECG alterations and were classified in functional class IV, which indicates incapacity to perform any activity without discomfort, including symptoms at rest. This classification is critical as it reflects a severe heart failure condition [48].

In the case of individuals infected with TcV, 66.7% exhibited major ECG alterations and were classified in functional class III. This class implies that less intense physical activities than usual cause symptoms, although the individual may be comfortable at rest [49,50].

Of the three individuals with mixed infections, all had altered BNP levels. Two of them exhibited major ECG alterations and were classified in functional class III. BNP is a crucial biomarker for assessing heart failure severity and serves as an important prognostic marker in the disease’s progression [51,52].

The data indicate that although most of the analyzed samples did not present a marked cardiac severity profile, a significant fraction of individuals with different DTUs exhibited relevant ECG alterations and functional classifications that suggest an increased risk. Understanding these profiles can help optimize treatment and improve the prognosis of affected individuals.

The *T. cruzi* genotyping approach used in this study follows one of the methodologies recommended by specialized reviews [53] and complies with the international consensus on DTU nomenclature [54]. This method is widely used [30,31,55,56] due to its high sensitivity in clinical samples — particularly in chronic patients with low parasitemia — as well as its cost-effectiveness and rapid interpretation. Direct genotyping allows for the detection of mixed infections and provides a more accurate assessment of parasite genetic diversity, whereas methods based on culture isolates may favor subpopulations adapted to in vitro conditions [53]. Although the multilocus PCR approach relies on multiple markers to distinguish closely related DTUs (such as TcII and TcVI), it employs well-established algorithms and strict controls, ensuring reliable results. The accuracy of this methodology has been validated by Sanger sequencing [19,31]. While alternative methods such as Sanger sequencing or next-generation sequencing (NGS) may overcome some limitations of PCR, they are significantly more complex and expensive. The approach used in this study offers an efficient combination of sensitivity, practicality, and cost-effectiveness, making it especially suitable for large-scale clinical analyses while maintaining reliability in DTU detection, including in mixed infections.This study has a limitation because it was not possible to amplify all genes essential for the molecular characterization of *T. cruzi*. Consequently, complete discrimination between DTUs did not occur in some samples, particularly those identified as TcII/TcVI. This limitation can be attributed to the low parasite load observed in samples from patients in the chronic phase of the disease, which makes it difficult to detect and amplify specific genetic markers. For statistical analysis purposes only, samples classified as TcII/TcVI were allocated to the TcII group, considering that this classification indicates an inconclusive genotyping result and that, in this sample set, the occurrence of TcII is epidemiologically more likely in the region [57]. Future studies with more comprehensive data may overcome these limitations and allow a more precise characterization of *T. cruzi* genotypes in chronic patients.

The data obtained in this study expand knowledge about the different strains of *Trypanosoma cruzi* and their distribution in endemic regions. In addition, the study confirmed, in a pioneering way, the presence of TcI and TcV in the North of Minas Gerais, as well as TcV in the Jequitinhonha Valley, highlighting the importance of continuing research on the genetic variability of the parasite. The characterization of the different genotypes contributes to a better understanding of the ecoepidemiology of Chagas disease, allowing the development of more effective control and prevention strategies.

## Supporting information

S1 Table General information about individuals included in the study (n = 80), parasite burden, and identification of *Trypanosoma cruzi* genotypes. (DOCX)
