# Supplementary material for: Genetic diversity of Trypanosoma cruzi in individuals with chronic Chagas disease in the Northern Minas Gerais and Vale do Jequitinhonha regions, Minas Gerais, Brazil
Source: PLoS Negl Trop Dis. 2025 Oct 15;19(10):e0013627. doi: 10.1371/journal.pntd.0013627 (PMC12548877; doi:10.1371/journal.pntd.0013627)
Supplement: S1 Table — (DOCX) [file pntd.0013627.s001.docx]

**Supplementary Table 1.** General information on individuals included in the study (n = 80), parasite load, identification of *Trypanosoma cruzi* genotypes and details of genotyped samples.

| **Samples** | **Municipalities** | **Age** | **Gender** | **Parasite load (average parasite/mL)** | **DTU** | **BNP** | **Ejection fraction** | **Functional class** | **ECG changes** |
| --- | --- | --- | --- | --- | --- | --- | --- | --- | --- |
| 1 | Berilo | 71 | Female | 0.41 | TcI | Unchanged | Normal | Class IV | Normal/ Minor changes |
| 2 | Berilo | 63 | Male | 2.87 | - | - | - | - | - |
| 3 | Berilo | 55 | Female | 1.38 | - | - | - | - | - |
| 4 | Berilo | 64 | Female | 7.38 | - | - | - | - | - |
| 5 | Berilo | 79 | Female | 4.72 | TcVI | Changed | Normal | Class III | Normal/ Minor changes |
| 6 | Berilo | 72 | Female | 1.43 | - | - | - | - | - |
| 7 | Berilo | 56 | Male | 2.10 | - | - | - | - | - |
| 8 | Berilo | 69 | Female | 5.32 | - | - | - | - | - |
| 9 | Berilo | 80 | Female | 2.34 | TcII/TcVI | Changed | Normal | Class II | Normal/ Minor changes |
| 10 | Bocaiúva | 67 | Female | 2.41 | - | - | - | - | - |
| 11 | Bocaiúva | 73 | Male | 5.49 | TcII/TcVI | Unchanged | Changed | Class II | NI |
| 12 | Bocaiúva | 81 | Female | 0.62 | TcI | Unchanged | Normal | Class III | Major changes |
| 13 | Brasília de Minas | 47 | Female | 1.27 | - | - | - | - | - |
| 14 | Brasília de Minas | 48 | Female | 27.80 | - | - | - | - | - |
| 15 | Carbonita | 82 | Female | 26.05 | TcII/TcVI | Unchanged | Normal | Class IV | Normal/ Minor changes |
| 16 | Carbonita | 54 | Female | 16.90 | TcV | Unchanged | Normal | Class I | Major changes |
| 17 | Carbonita | 89 | Female | 0.06 | - | - | - | - | - |
| 18 | Carbonita | 71 | Female | 0.04 | - | - | - | - | - |
| 19 | Carbonita | 67 | Female | 0.93 | - | - | - | - | - |
| 20 | Chapada do Norte | 65 | Female | 8.64 | - | - | - | - | - |
| 21 | Claro dos Poções | 60 | Female | 0.03 | - | - | - | - | - |
| 22 | Claro dos Poções | 76 | Female | 7.07 | TcVI | Changed | Normal | Class IV | Major changes |
| 23 | Claro dos Poções | 78 | Female | 1.03 | - | - | - | - | - |
| 24 | Claro dos Poções | 69 | Female | 7.07 | - | - | - | - | - |
| 25 | Claro dos Poções | 71 | Female | 2.78 | - | - | - | - | - |
| 26 | Francisco Badaró | 68 | Female | NI | - | - | - | - | - |
| 27 | Francisco Badaró | 71 | Male | 21.95 | - | - | - | - | - |
| 28 | Francisco Badaró | 96 | Female | 0.00 | - | - | - | - | - |
| 29 | Francisco Badaró | 54 | Male | 0.06 | TcVI | Unchanged | Normal | Class III | Normal/ Minor changes |
| 30 | Francisco Badaró | 71 | Male | 6.47 | - | - | - | - | - |
| 31 | Francisco Badaró | 80 | Female | NI | TcII/TcVI | Unchanged | NI | Class II | Normal/ Minor changes |
| 32 | Francisco Sá | 76 | Female | 42.98 | - | - | - | - | - |
| 33 | Francisco Sá | 34 | Female | 0.18 | TcV + TcII | Changed | Normal | Class II | Major changes |
| 34 | Fruta de Leite | 51 | Female | 1.04 | - | - | - | - | - |
| 35 | Fruta de Leite | 38 | Male | 0.80 | - | - | - | - | - |
| 36 | Fruta de Leite | 58 | Female | 0.36 | TcII/TcVI | Unchanged | Normal | Class I | Normal/ Minor changes |
| 37 | Fruta de Leite | 61 | Female | 6.30 | TcII/TcVI | Unchanged | Normal | Class III | Normal/ Minor changes |
| 38 | Fruta de Leite | 72 | Female | 12.71 | - | - | - | - | - |
| 39 | Fruta de Leite | 50 | Female | 32.25 | - | - | - | - | - |
| 40 | Janaúba | 61 | Male | 667.00 | TcII | Unchanged | Normal | Class I | Normal/ Minor changes |
| 41 | Janaúba | 50 | Male | 9.26 | - | - | - | - | - |
| 42 | Janaúba | 54 | Female | 23.90 | TcII/TcVI | Unchanged | Normal | Class II | Normal/ Minor changes |
| 43 | Janaúba | 61 | Male | 173.00 | - | - | - | - | - |
| 44 | Janaúba | 51 | Female | 30.03 | TcII/TcVI | Unchanged | Normal | Class IV | Major changes |
| 45 | Janaúba | 80 | Female | 0.09 | - | - | - | - | - |
| 46 | Jenipapo de Minas | 91 | Female | 476.50 | TcV | Unchanged | Normal | Class III | Major changes |
| 47 | Leme do Prado | 64 | Male | 7.90 | TcII/TcVI | Unchanged | Changed | Class I | Major changes |
| 48 | Leme do Prado | 84 | Female | 19.15 | TcVI | Changed | Normal | Class IV | Major changes |
| 49 | Minas Novas | 71 | Female | 8.90 | - | - | - | - | - |
| 50 | Minas Novas | 57 | Female | 7.55 | - | - | - | - | - |
| 51 | Minas Novas | 65 | Male | 0.33 | - | - | - | - | - |
| 52 | Minas Novas | 65 | Female | 13.50 | TcII | Unchanged | Normal | Class I | Normal/ Minor changes |
| 53 | Minas Novas | 73 | Male | 41.60 | TcI | Unchanged | Normal | Class IV | Normal/ Minor changes |
| 54 | Monte Azul | 43 | Female | 23.85 | - | - | - | - | - |
| 55 | Pai Pedro | 65 | Female | 2.92 | TcVI | Changed | Normal | Class I | Normal/ Minor changes |
| 56 | Pai Pedro | 50 | Female | 19.20 | TcII | Unchanged | Normal | Class IV | Major changes |
| 57 | Pai Pedro | 74 | Female | 3.46 | TcII/TcVI | Unchanged | Normal | Class II | Normal/ Minor changes |
| 58 | Rio Pardo de Minas | 56 | Male | 15.40 | TcII/TcVI | Unchanged | Normal | Class I | Normal/ Minor changes |
| 59 | São Francisco | 49 | Female | 1.83 | - | - | - | - | - |
| 60 | São Francisco | 60 | Female | 6.31 | TcII/TcVI | Unchanged | Normal | Class IV | Major changes |
| 61 | São Francisco | 80 | Male | 96.55 | - | - | - | - | - |
| 62 | São Francisco | 65 | Female | 7.09 | - | - | - | - | - |
| 63 | São Francisco | 54 | Male | 1.91 | - | - | - | - | - |
| 64 | São Francisco | 58 | Male | 0.74 | TcII/TcVI | Unchanged | Normal | Class III | Normal/ Minor changes |
| 65 | São Francisco | 47 | Female | 10.34 | TcI + TcV + TcII/ TcVI | Changed | Normal | Class III | Major changes |
| 66 | São Francisco | 48 | Female | 0.00 | - | - | - | - | - |
| 67 | São Francisco | 75 | Female | 2.40 | TcII/TcVI | Unchanged | Normal | Class II | Normal/ Minor changes |
| 68 | São Francisco | 52 | Female | 2.42 | - | - | - | - | - |
| 69 | Turmalina | 78 | Female | 28.60 | TcV | Unchanged | Normal | Class III | Normal/ Minor changes |
| 70 | Turmalina | 75 | Female | 9.79 | TcV + TcVI | Changed | Normal | Class III | Normal/ Minor changes |
| 71 | Turmalina | 72 | Male | 0.11 | TcII | Unchanged | Changed | Class IV | Major changes |
| 72 | Turmalina | 71 | Male | 0.96 | - | - | - | - | - |
| 73 | Turmalina | 70 | Female | 24.70 | TcII/TcVI | Unchanged | Normal | Class I | Normal/ Minor changes |
| 74 | Turmalina | 57 | Male | 0.65 | TcI | Unchanged | Normal | Class IV | Major changes |
| 75 | Turmalina | 62 | Male | 12.21 | TcI | Unchanged | Normal | Class I | Major changes |
| 76 | Turmalina | 72 | Female | 8.87 | TcII/TcVI | Unchanged | Normal | Class III | Major changes |
| 77 | Ubaí | 62 | Female | 13.72 | TcII/TcVI | Unchanged | Normal | Class IV | Major changes |
| 78 | Verdelândia | 53 | Female | 7.71 | - | - | - | - | - |
| 79 | Verdelândia | 66 | Female | 0.69 | - | - | - | - | - |
| 80 | Verdelândia | 41 | Female | 1.51 | - | - | - | - | - |

NI: Not Informed.
